# Intersectionality and discriminatory practices within mental health care

**DOI:** 10.1186/s13010-024-00159-7

**Published:** 2024-06-28

**Authors:** Mirjam Faissner, Anne-Sophie Gaillard, Georg Juckel, Amma Yeboah, Jakov Gather

**Affiliations:** 1 Department of Psychiatry, Psychotherapy and Preventive Medicine, LWL University Hospital, Ruhr University Bochum, Bochum, Germany; 2 Institute of the History of Medicine and Ethics in Medicine, Charité - Universitätsmedizin Berlin, Thielallee 71, 14195 Berlin, Germany; 3 Institute for Medical Ethics and History of Medicine, Ruhr University Bochum, Bochum, Germany; 4 Private Practice for Psychiatry, Psychotherapy, and Psychodynamic Supervision, Cologne, Germany

## Introduction

Through this article collection, we invite readers to get acquainted with intersectionality as a tool to help us challenge mental health care practices that reproduce social injustice. Social injustice describes how the set-up of societies, including social processes and institutions, can disproportionally disadvantage members of marginalized social groups, thereby unjustly constraining their possibilities for flourishing [1]. The stigmatizing effects of mental health diagnoses, which still, in many contexts, shape a person’s conditions of flourishing, are well known [2]. Yet it is less considered that when a person receives a mental health diagnosis, that person already occupies a social position characterized by various markers of identity that constrain and enable their possibilities [3]. We intuitively see that the situation of a White high school teacher with depression differs from the situation of a trans woman of color with anxiety. Yet what difference does this difference make? And how can we approach such differences within mental health care from a social justice perspective?

Intersectionality is at the core for understanding the lived reality of experiencing mental illness in societies shaped by social systems of power and oppression, such as ableism, sexism, classism, cis-heteronormativism or Whiteness. It has a long tradition within social justice work, research and activism. Ideas of intersectionality can be dated back to a famous speech by Sojourner Truth [4] who, at the Ohio Women’ Convention in 1851 at Akron, Ohio, urged her feminist audience to acknowledge her identity both as a Black person and a woman. Since then, intersectionality has been practiced, further developed and reflected upon by various people and groups, such as Mary Church Terrell [5], the Combahee River Collective [6], bell hooks [7] or Patricia Hill Collins [8]. In 1989, the legal theorist Kimberlé Crenshaw [9] coined ‘intersectionality’ as a metaphor, and introduced it into the academic realm, where it has encouraged justice-oriented research projects in many disciplines, including public health [10] and psychology [11].

Intersectionality describes a combined analysis and practice in the pursuit of social justice [8]. It highlights that different systems of power and oppression are inherently interlinked, and that experiences of discrimination are complex and occur in simultaneity [12]. Thus, a person’s challenges to navigate our social world – including the health care system – as a trans woman, *and* as a woman of color *and* as a person with anxiety cannot be reduced to any one axis of discrimination, and need to be addressed inclusively and under consideration of the complexities posed by co-constitutive power structures.

## Bochum INSIST summer school

The articles collected here result from an interdisciplinary and international Summer School “Intersectionality as a tool to prevent structural discrimination. Ethical, legal and social aspects of strategies of anti-discrimination in mental health care” from September 5–9 2022 in Bochum, Germany. The summer school was organized by Mirjam Faissner, Jakov Gather and Georg Juckel, and hosted by the Department of Psychiatry, Psychotherapy and Preventive Medicine, LWL University Hospital at Ruhr University Bochum. It was funded by the German Federal Ministry of Education and Research (grant number: 01GP2188). The summer school aimed at bringing scholars from different fields together to understand the multifaceted and complex structures of power, various forms of discriminatory practices, and to discuss strategies for a more inclusive and social justice-based practice, as well as forms of anti-discrimination within mental health care.

Psychiatry, as an institution, has a long history of pathologizing and marginalizing people based on different forms of constructed ‘ab-normality’. For this reason, the central motivation of the initiators of the INSIST summer school, as part of the psychiatric institution, was to stimulate change from within. Opening a space for critique and self-reflection within the institution was an important aim of the summer school. To that end, we invited young scholars and senior researchers from psychiatry, philosophy, psychology, musical therapy, medical ethics, sociology, educational sciences, social work, disability studies and epidemiology. During the summer school, participants engaged with each other in workshops and paper presentations on the topics of intersectionality, heteronormativism, racism, transphobia, ageism, and human rights. Expert lectures were held by Claudia Bernard (Professor of Social Work at Goldsmith University London), Theresia Degener (Professor of Law and Disability Studies at Protestant University of Applied Sciences of the Rheinland-Westfalen-Lippe), Ulrike Kluge (Professor of Psychological and Medical Integration and Migration Studies at Charité University Medicine Berlin), Stephani Hatch (Professor of Sociology and Epidemiology at King’s College London), Katharine Jenkins (lecturer in Philosophy at University of Glasgow) and Amma Yeboah (psychodynamic supervisor, specialist and senior consultant in psychiatry and psychotherapy). During the summer school, participants were invited to collaborate in interdisciplinary writing groups with shared research interests. The groups were designed as a space to delve into a topic and to collaboratively develop article ideas. Three of the original articles published here were conceptualized during these workshops.

## Overlook on the articles from this collection

The articles in this collection use intersectionality as an *analytic lens* [13, 14], as a *critical practice tool* [15] and as a *reflection tool* [16, 17].

Funer [13] provides an empirically informed argument to increase the use of intersectional frameworks within mental health research, policy and practice. Starting from a public mental health perspective, Funer notes the potential of intersectionality to understand how mental health disparities across social groups are related to co-constitutive and interrelated systems of structural oppression, including racism, sexism, classism, ableism, and homophobia, leading to unique positions of disadvantage that affect mental health. Funer argues that mental health professionals may be better able to support people experiencing mental health complaints if they acknowledge the social and structural causes of mental distress and consider the distinct positions of intersectional disadvantage that people experiencing mental distress occupy within structural systems of discrimination.

Langmann and Wessel [14] use intersectionality to critically interrogate the concept of ‘successful aging’, which has prominently been used in gerontology, a field with an important overlap with mental health care. More specifically, Langmann and Wessel, based on several examples from mental health care, identify ageist and ableist biases in the discourse on successful aging. In one case analysis, they show how the cumulation of classism, sexism, and racism leads to increased risks of depression in poor Black women, a risk often not adequately considered in the literature on good aging. The authors argue for a new approach to aging that rejects a universal definition of ‘aging well’ and, by contrast, considers the diverse experiences of ‘aging well’ that are influenced by race, gender, socio-economic status, and disabilities.

Faissner and colleagues [15] consider intersectionality as a tool for clinical ethicists. First, they provide reasons to use intersectionality in clinical ethics consultations: According to their normative argument, clinical ethicists are committed to pursuing social justice in health care and are well-positioned to do so. According to the authors’ epistemic argument, power structures influence both ethical conflicts in clinical practice and dynamics within clinical ethics consultations, so that not considering these influences leads to information losses and potentially insufficient or mistaken ethical analyses. Based on a concrete case example from mental healthcare, the authors explicate how intersectionality can contribute to addressing structural discrimination in clinical ethics consultations. To this end, they critically review existing approaches for clinical ethics consultants to address structural racism in clinical ethics consultations and extend them by intersectional considerations.

Epistemic appropriation is a process by which a marginalized community is harmed if the knowledge and ideas they have developed are detached from the originating authors and then misdirected through using them for goals not directed towards the originating community. Myerscough and colleagues [16] analyze how mental health research threatens to epistemically appropriate knowledge developed in trans communities. They provide an in-depth analysis of how concerns around detransition and transition regret have been misdirected for anti-trans goals. In line with intersectionality as a tool for critical reflection, the authors urge researchers to critically question their own motivation, subject position, and relationship to the trans community when engaging in trans mental health research.

In her original commentary, Villar [17] utilizes an interpretation of Charlotte Perkins Gilman’s 1892 gothic short story “The Yellow Wallpaper” to draw attention to socially unjust practices in mental health care practice and research. In the story, a White young woman diarist suffering from post-partum depression with psychotic features chronicles her experience of the “Rest Cure” provided by her physician husband. Following an intersectional analysis of the social positioning and power dynamics of the characters in the short story, Villar employs the narrative as a starting point to critically reflect on the representation of marginalized service users in clinical research.

### Outlook

The articles in this collection draw attention to different forms of social injustice, how they affect mental health, health care practices and research, and what could be done to address them. By creating spaces for the topic of social injustice, by acknowledging it, and by questioning the existing power structures and hierarchies that shape our professional care, the contributors of this collection already practice intersectionality. We invite readers who wish for a more inclusive mental health care practice to join this process as a joint effort. Together, we can think of intersectionality as a vision, as a forward-looking collaborative practice of improving mental healthcare in our shared social world.
